# Characterization of *Arabidopsis thaliana* Hydroxyphenylpyruvate Reductases in the Tyrosine Conversion Pathway

**DOI:** 10.3389/fpls.2018.01305

**Published:** 2018-09-05

**Authors:** Jing-Jing Xu, Xin Fang, Chen-Yi Li, Qing Zhao, Cathie Martin, Xiao-Ya Chen, Lei Yang

**Affiliations:** ^1^Shanghai Key Laboratory of Plant Functional Genomics and Resources, Plant Science Research Center, Shanghai Chenshan Botanical Garden, Shanghai, China; ^2^National Key Laboratory of Plant Molecular Genetics, CAS Center for Excellence in Molecular Plant Sciences, Shanghai Institute of Plant Physiology and Ecology, Chinese Academy of Sciences, Shanghai, China; ^3^John Innes Centre, Norwich Research Park, Norwich, United Kingdom

**Keywords:** hydroxyphenylpyruvate reductase, 4-hydroxyphenyllactic acid, tyrosine, rosmarinic acid, *Arabidopsis thaliana*, secondary metabolism

## Abstract

Tyrosine serves as a precursor to several types of plant natural products of medicinal or nutritional interests. Hydroxyphenylpyruvate reductase (HPPR), which catalyzes the reduction of 4-hydroxyphenylpyruvic acid (pHPP) to 4-hydroxyphenyllactic acid (pHPL), has been shown to be the key enzyme in the biosynthesis of rosmarinic acid (RA) from tyrosine and, so far, HPPR activity has been reported only from the RA-accumulating plants. Here, we show that HPPR homologs are widely distributed in land plants. In *Arabidopsi*s *thaliana*, which does not accumulate RA at detectable level, two homologs (HPPR2 and HPPR3) are functional in reducing pHPP. Phylogenetic analysis placed HPPR2 and HPPR3 in two separate groups within the HPPR clade, and HPPR2 and HPPR3 are distinct from HPR1, a peroxisomal hydroxypyruvate reductase (HPR). *In vitro* characterization of the recombinant proteins revealed that HPPR2 has both HPR and HPPR activities, whereas HPPR3 has a strong preference for pHPP, and both enzymes are localized in the cytosol. *Arabidopsis* mutants defective in either *HPPR2* or *HPPR3* contained lower amounts of pHPL and were impaired in conversion of tyrosine to pHPL. Furthermore, a loss-of-function mutation in tyrosine aminotransferase (TAT) also reduced the pHPL accumulation in plants. Our data demonstrate that in *Arabidopsis* HPPR2 and HPPR3, together with TAT1, constitute to a probably conserved biosynthetic pathway from tyrosine to pHPL, from which some specialized metabolites, such as RA, can be generated in specific groups of plants. Our finding may have broad implications for the origins of tyrosine-derived specialized metabolites in general.

## Introduction

Tyrosine is a precursor to numerous plant natural products such as tocopherols, plastoquinone, and the specialized (or secondary) metabolites of dhurrin, betalains, benzylisoquinolines, and rosmarinic acid (RA), which have diverse functions in plant growth, development, and adaptation. Furthermore, many of the tyrosine-derived compounds are of high value in medicine and human nutrition. However, despite their substantial benefits for human health and their important functions in the physiology of plants, our understanding of the enzymes involved in tyrosine metabolism remains rudimentary.

**FIGURE 1:**
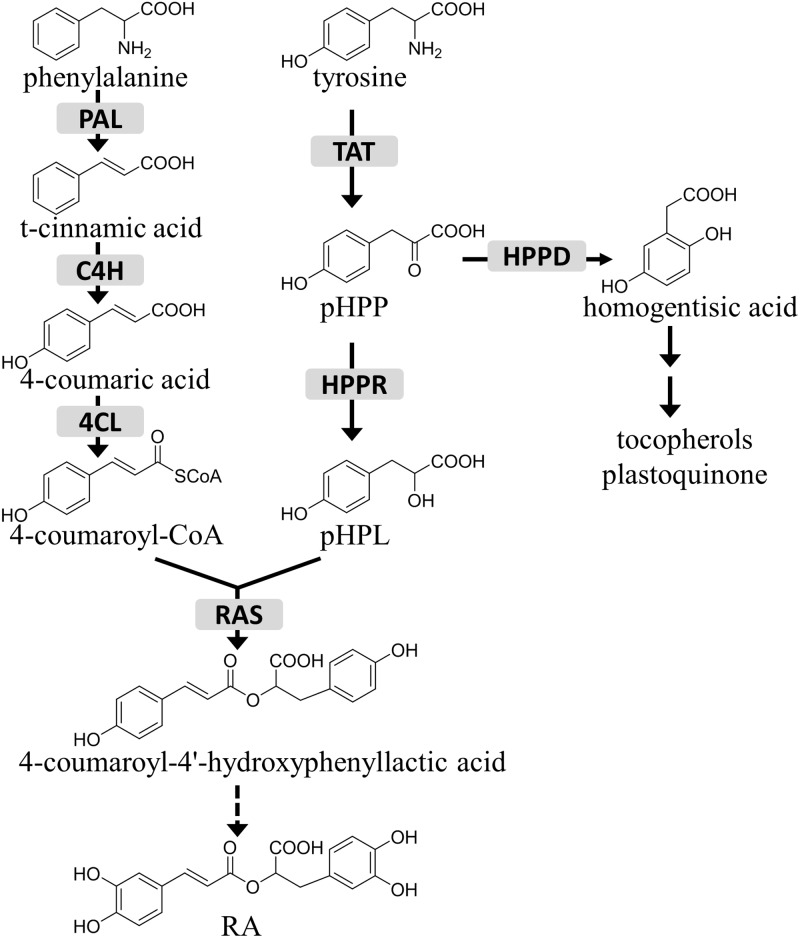
The role of HPPR in the tyrosine conversion pathway. TAT, tyrosine aminotransferase; HPPR, hydroxyphenylpyruvate reductase; HPPD, 4-hydroxyphenylpyruvate dioxygenase; RAS, rosmarinic acid synthase; PAL, phenylalanine ammonia-lyase; C4H, cinnamic acid 4-hydroxylase; 4CL, 4-coumaric acid CoA-ligase; pHPP, 4-hydroxyphenylpyruvic acid; pHPL, 4-hydroxyphenyllactic acid; RA, rosmarinic acid.

Tyrosine aminotransferase (TAT) catalyzes the reversible transamination from tyrosine to form 4-hydroxyphenylpyruvic acid (pHPP), an initial step in the biosynthesis of many tyrosine-derived metabolites (**Figure 1**). pHPP is converted by 4-hydroxyphenylpyruvate dioxygenase (HPPD) to homogentisic acid, the aromatic precursor of tocopherols and plastoquinone, which are essential for photosynthetic organisms (Amesz, 1973; Norris et al., 1995; Munne-Bosch and Alegre, 2002). In addition, in certain groups of plants pHPP is used for the synthesis of RA, a caffeic acid ester of 3, 4-dihydroxyphenyllactic acid. While the 3, 4-dihydroxyphenyllactic acid part of the RA molecule is derived from tyrosine, the caffeic acid moiety comes from the general phenylpropanoid pathway via 4-coumaroyl-CoA, which is synthesized from phenylalanine by phenylalanine ammonia-lyase (PAL), cinnamic acid 4-hydroxylase (C4H), and 4-coumaric acid CoA-ligase (4CL) (Petersen et al., 1993). The general phenylpropanoid pathway also provides precursors for the biosynthesis of flavonoids, lignins, and coumarins, as well as for the aromatic volatiles (Hahlbrock and Scheel, 1989; Martin et al., 2001; Naoumkina et al., 2010; Widhalm and Dudareva, 2014). It has been reported that pHPP is reduced to 4-hydroxyphenyllactic acid (pHPL) by hydroxyphenylpyruvate reductase (HPPR), which was thought to be the first step committed to RA production (Kim et al., 2004).

HPPR belongs to the family of D-isomer-specific 2-hydroxyacid dehydrogenases (Grant, 1989). Enzymes in this family catalyze the reversible reduction of 2-ketoacids into D-2-hydroxyacids using NADH or NADPH as a coenzyme. The HPPR enzyme was first purified from cell cultures of *Coleus blumei* (*Plectranthus scutellarioides*), a garden plant in the family Lamiaceae (Petersen and Alfermann, 1988). Later, it was reported that HPPR shows the highest affinity to pHPP, but will also accept 3, 4-dihydroxyphenylpyruvic acid and 3-methoxy-4-hydroxyphenylpyruvic acid as substrates (Hausler et al., 1991). Putative *HPPR* genes have been identified in *Salvia* species (Xiao et al., 2011; Barberini et al., 2013; Wang et al., 2015, 2017), many of which are medicinal plants widely used in China.

The two precursors of the RA molecule, 4-coumaroyl-CoA and pHPP, are widely distributed in land plants. By contrast, RA has limited distributions. In angiosperms RA is frequently found in plants of the families Lamiaceae and Boraginaceae, with occasional reports of its presence in other mono- and dicotyledonous families. RA also occurs in plants of hornworts and the fern families Blechnaceae and Dennstaedtiaceae (Petersen and Simmonds, 2003; Petersen et al., 2009; Petersen, 2013). RA synthase (RAS), first identified from *Coleus blumei*, catalyzes the condensation of pHPL and 4-coumaroyl-CoA, followed by hydroxylation to form RA (Petersen, 1991; Petersen et al., 1993). RAS belongs to the BAHD acyltransferase family (Berger et al., 2006) and likely emerged from its evolutionary progenitor hydroxycinnamoyl-CoA:shikimate hydroxycinnamoyl transferase (HCT), a more conserved enzyme ubiquitously present in land plants (Weng et al., 2012). Phylogenetic analyses showed that RASs are separated from HCTs and, notably, the RAS subgroup includes members of the RA-accumulating plant species only (Petersen et al., 2009). We were interested to determine whether the taxa-specific distribution of RA is due to the similarly limited occurrence of HPPR or RAS. In this investigation, we discovered that the HPPR homologs are commonly present in land plants, and that the *Arabidopsis* genome contains three *HPPR* genes, *HPPR2* (At1g79870), *HPPR3* (At1g12550), and *HPPR4* (At2g45630), of which *HPPR2* and *HPPR3* encode functional HPPRs. Data from *in vitro* enzyme assay and mutant analysis showed that TAT and HPPR catalyze two sequential steps in the conversion of tyrosine to pHPL in *Arabidopsis*, suggesting that HPPR is part of a more widespread biosynthetic pathway from which RA biosynthesis has branched in specific groups of plants.

## Materials and Methods

### Plant Material

All *Arabidopsis thaliana* lines used in this study were in the Columbia-0 (Col-0) background. The T-DNA insertional mutants of GK-471A09 (*hppr2-1*), SALK_143689 (*hppr3-1*), SALK_019014 (*hppr3-2*), SALK_045398 (*tat1-1*), SALK_141402 (*tat1-2*), SALK_052382 (*tat2-1*), and GK-471E06 (*tat2-2*) were obtained from the Nottingham *Arabidopsis* Stock Center (NASC). Homozygous plants were identified by genomic PCR, and the gene expression levels were examined by qRT-PCR. Primers used in this investigation are listed in **Supplementary Table S1**.

The *hppr2-c1* mutant was generated using CRISPR/Cas9-mediated genome editing. Golden Gate cloning (Weber et al., 2011) was used to generate the constructs designed to create a large deletion in *HPPR2* gene using two sgRNAs, and the cassettes were driven by AtU6-26 promoter. Level 1 constructs pICSL11059::35S::hptII, pICSL11049::AtUbi10::Cas9, pICSL11019::AtUbi5::turboGFP, pICH47761::AtU6-26p::sgRNA1, pICH47772::AtU6-26p::sg RNA2, and the linker pICH41800 were assembled into the level 2 vector pAGM4723 as described (Weber et al., 2011). The construct was transformed into *Arabidopsis* through floral dipping (Clough and Bent, 1998).

pICH47761 (Addgene plasmid # 48003), pICH47772 (Addgene plasmid # 48004), pICH41800 (Addgene plasmid # 48020), and pAGM4723 (Addgene plasmid # 48015) were gifts from Sylvestre Marillonnet. pICSL11059 (Addgene plasmid # 68263) was a gift from Nicola Patron.

### Phylogenetic Analysis

The protein sequence of *Coleus blumei* HPPR was obtained from NCBI, the HPPR sequences of *Selaginella moellendorffii*, *Picea sitchensis*, and *Taxus baccata* were from the PLAZA project (Van Bel et al., 2018), and the others were obtained from the Ensembl database. The protein sequences were aligned using ClustalX (Thompson et al., 1997), and the phylogenetic tree was generated using MEGA 7.0 (Kumar et al., 2016) by the neighbor-joining method (1000 bootstrap replication, pair-wise deletion, and Poisson correction).

### Analysis of pHPL

For feeding experiments, plants were germinated and grown on the Murashige–Skoog (MS) agar medium. Fourteen-day-old seedlings were transferred to MS liquid medium containing 250 μM ^13^C_6_-tyrosine (Cambridge Isotope Laboratories). Samples were harvested 24 h later, frozen in liquid nitrogen, and freeze-dried. Ten milligram of freeze-dried samples were extracted with 300 μL of 75% methanol containing 0.5 mg L^−1^ 4-methylumbelliferone (internal standard) in a sonicator bath for 2 h. The supernatant was hydrolyzed with 1 M HCl at 90°C for 1 h.

pHPL was detected by liquid chromatography-multiple reaction monitoring-mass spectrometry (LC-MRM-MS) in negative ionization mode using an Agilent 1260 HPLC and 6460 Triple Quadrupole LC/MS system. An Agilent XDB-C18 column (4.6 × 250 mm, 5 μm particles) was used at 30°C, flow rate of 1 mL/min and with an 18-min linear gradient of 5–70% acetonitrile in 0.1% formic acid. Product ion spectra were used to determine MRM transitions of 187 > 169 for labeled pHPL, 181 > 163 for unlabeled pHPL, and 175 > 133 for 4-methylumbelliferone, respectively.

### Heterologous Expression and Purification of Recombinant Proteins

The coding regions of *HPR1*, *HPPR2*, *HPPR3*, and *HPPR4* were amplified from *Arabidopsis* complementary DNAs and cloned into the Gateway vector pDEST17 to yield the *E. coli* expression constructs. The plasmids were introduced into *E. coli* strain Rose-gami B (DE3). Protein production was induced with isopropyl β-D-1-thiogalactopyranoside (0.1 mM) for 20 h at 16°C. The cells were lysed by sonication, and the recombinant proteins were purified with Ni-NTA His-Bind resin (Thermo). The isolated enzymes were further desalted on PD-10 columns (GE Healthcare) equilibrated with buffer (10 mM KH_2_PO4/K_2_HPO4, PH 7.0, 30% glycerol, and 2 mM β-mercaptoethanol). Protein concentration was determined using Bradford reagent (Bio-Rad).

### Enzyme Assays

Assays (250 μL) contained 100 mM KH_2_PO4/K_2_HPO4 (PH 7.0), 40 μM ascorbate, 4 mM DTT, 1 mM pHPP (or other substrate as indicated specifically), 2 mM NADH or NADPH, and 1 to 5 μg of recombinant enzyme, as described (Petersen and Alfermann, 1988). Reactions were performed for 60 min at 30°C and stopped by addition of 25 μL 6 M HCl, the resulted products were extracted three times with ethyl acetate, dried in vacuo, and redissolved in 15% methanol. For kinetics measurements pHPP, phenylpyruvic acid or hydroxypyruvic acid were used at concentrations ranging from 30 μM to 7 mM. The reaction time was 5–20 min. Reaction products were quantified by MRM in negative ion mode for all assays. Kinetic parameters of *V*_max_ and *K*_m_ were determined by non-linear regression analysis with Hill type equations using Origin 8.

### Quantitative Reverse Transcription PCR (qRT-PCR)

Total RNAs was extracted from 7-day-old seedlings, 4-week-old rosette leaves, stems and leaves of 5-week-old plants, and opened flowers using the RNAiso Plus reagent (Takara Biotechnology, Dalian, China). RNA (∼500 ng) was used for cDNA synthesis using a PrimeScript RT reagent Kit with gDNA Eraser (Takara). qRT-PCR was carried out using Mastercycler ep Realplex2 (Eppendorf) using the SYBR Green reagent (Takara). For quantification of *HPR1*, *HPPR2*, *HPPR3*, and *HPPR4* transcript levels, the pDONR207 vectors carrying *HPR1*, *HPPR2*, *HPPR3*, and *HPPR4* were serially diluted 10-fold to generate standard curves. The *PP2AA3* (*At1g13320*) gene was used as an internal reference (Czechowski et al., 2005).

### GUS Analysis

Promoter regions of *HPPR2* (1004 bp), *HPPR3* (1239 bp), and *HPPR4* (914 bp) were amplified by PCR from *Arabidopsis* Col-0 genomic DNA using the primers as listed (**Supplementary Table S1**), followed by cloning into the vector pGWB533 (Nakagawa et al., 2007) using Gateway technology. The reporter genes were then transferred to *Arabidopsis*. For each construct, three independent transformant lines were selected for GUS staining.

The histochemical GUS assays were performed following the protocol of Jefferson et al. (1987). The plant material was incubated at 37°C in the dark for 12–18 h in staining buffer (0.5 mg mL^−1^ 5-bromo-4-chloro-3-indolyl-β-D-glucuronide in 0.1 M Na_2_HPO4, pH 7.0, 10 mM Na_2_EDTA, 0.5 mM potassium ferricyanide, 0.5 mM potassium ferrocyanide, and 0.1% Triton X-100). Samples were washed with 70% ethanol and visualized using a dissecting microscope (OLYMPUS SZX7).

### Subcellular Localization

cDNAs of *HPR1*, *HPPR2*, *HPPR3*, and *HPPR4* were subcloned into pK7WGF2 using Gateway technology. *Arabidopsis* protoplasts prepared from mesophyll cells were transfected with the plasmids. After 16–20 h of transfection, protoplasts were observed using an Olympus FV10i confocal laser scanning microscope. Excitation wavelengths were 473 nm for GFP and 635 nm for chlorophyll. Emissions were collected from 490 to 540 nm for GFP, and 660 to 710 nm for chlorophyll.

## Results

### Phylogenetic Analysis of HPPRs and HPRs

**FIGURE 2:**
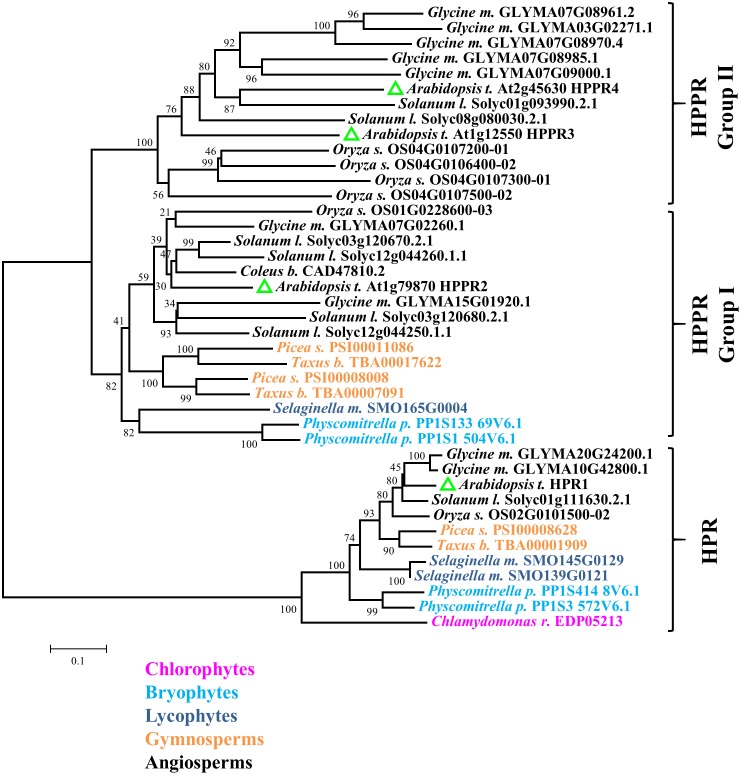
HPPR homologs are widely distributed in land plants. The neighbor-joining method was used to perform a phylogenetic analysis of HPPRs and HPRs from *Chlamydomonas reinhardtii*, *Physcomitrella paten*s, *Selaginella moellendorffii*, *Picea sitchensis*, *Taxus baccata*, *Oryza sativ*a, *Glycine max*, *Arabidopsis thaliana*, *Solanum lycopersicum*, and *Coleus blumei*.

A BLAST search of the *Arabidopsis* genome revealed that the predicted proteins encoded by At1g79870, At1g12550, and At2g45630 share 76, 48, and 45% identities with the HPPR (CAD47810.2) from *Coleus blumei* at the amino acid sequence level. However, At1g79870 and At1g12550 have been reported to be hydroxypyruvate reductases (HPRs), which reduce hydroxypyruvic acid to D-glyceric acid, a key step of the photorespiratory cycle (Timm et al., 2008, 2011). To examine the distribution of putative HPPRs in plants, we searched for HPR and HPPR homologs from taxonomically diverse species (**Figure 2**). A phylogenetic tree of these predicted proteins showed two major clades. One, containing the *Arabidopsis* peroxisomal hydroxypyruvate reductase1 (HPR1) and designated as the HPR clade, includes representatives from all Viridiplantae species surveyed, including *Chlamydomonas reinhardtii*, *Physcomitrella paten*s, *Selaginella moellendorffii*, *Picea sitchensis*, *Taxus baccata*, and all angiosperms investigated. The other clade, named HPPR because it includes HPPR from *Coleus blumei*, can be divided further into two groups. Group I includes members of all land plants analyzed, and group II is angiosperm-specific. At1g79870 belongs to group I, whereas At1g12550 and At2g45630 belong to group II. We named these predicted proteins HPPR2, HPPR3, and HPPR4, respectively. The phylogenetic analysis suggested that the HPPRs likely evolved in the ancestral land plants from the HPR clade.

### HPPR2 and HPPR3 Are Functional in Reducing pHPP

**FIGURE 3:**
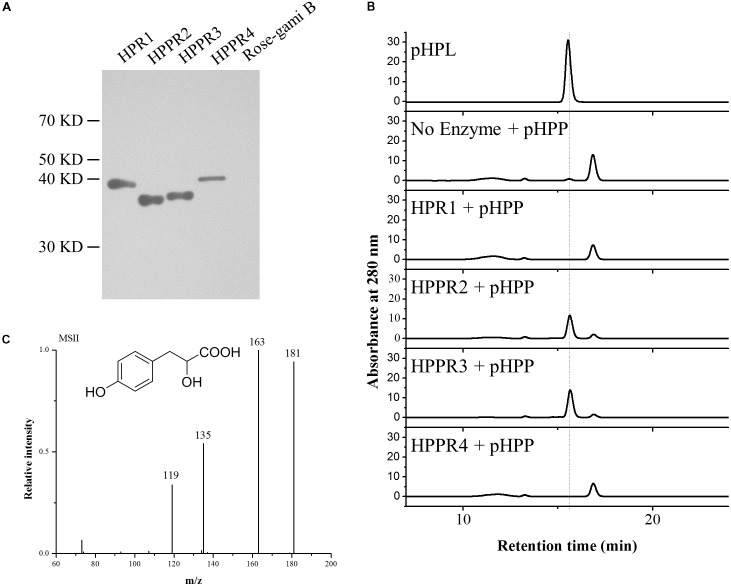
HPPR2 and HPPR3 have HPPR activity *in vitro*. **(A)** Western blot analysis of purified recombinant proteins expressed in *E. coli* using anti-His antibody. **(B)** HPLC profiles of the pHPL standard and the reaction products after incubation of no enzyme, HPR1, HPPR2, HPPR3, and HPPR4 with pHPP as substrate. **(C)** Mass spectrum and structure of pHPL.

To determine their catalytic activities, the three new HPPR homologs HPPR2, HPPR3, and HPPR4, as well as HPR1 from *Arabidopsis*, were expressed in *Escherichia coli*. The purified recombinant enzymes (**Figure 3A**) were incubated with pHPP as a substrate. While HPR1 and HPPR4 did not exhibit HPPR activity under our assay conditions, HPPR2 and HPPR3 converted pHPP to a product (**Figure 3B**), which was confirmed to be 4-hydroxyphenyllactic acid (pHPL) by liquid chromatography coupled with tandem mass spectrometry (LC-MS/MS) (**Figure 3C**).

To further examine the activities of HPR1 and the three HPPR homologs of *Arabidopsis*, their substrate preferences were tested using pHPP and hydroxypyruvic acid as the substrates. In addition, phenylpyruvic acid was also tested to investigate the possible roles of these enzymes in phenylalanine metabolism as well as their catalytic promiscuity. HPR1 showed a high apparent catalytic efficiency (*k*_cat_/*K*_m_) in reducing hydroxypyruvic acid (**Table 1**). HPPR2 had the highest affinity for pHPP, although its highest catalytic efficiency was detected with hydroxypyruvic acid. The apparent *K*_m_ value of HPPR3 for pHPP was lower than the values for phenylpyruvic acid and hydroxypyruvic acid, indicating that HPPR3 has the highest affinity for pHPP. Consequently, HPPR3 exhibited the highest efficiency in reducing pHPP to pHPL. HPPR4 showed little activity with any of the three substrates assayed. These results indicated that HPPR2 and HPPR3 are promiscuous toward pyruvic acids; while both have a clear HPPR activity, HPPR3 preferentially acts on pHPP and therefore is likely a more specific HPPR.

**Table 1:** Kinetic parameters of HPR1 and HPPRs.

Enzyme	Substrate	Cofactor	*K*_m_ (mM)	*k*_cat_ (min^−1^)	*k*_cat_/*K*_m_ (min^−1^ mM^−1)^
HPR1	pHPP	Activity was not detectable			
	Phenylpyruvic acid	Activity was not detectable			
	Hydroxypyruvic acid	NADH	1.53 ± 0.72	207.40 ± 5.03	153.92 ± 62.41
HPPR2	pHPP	NADPH	0.80 ± 0.16	7.33 ± 0.51	9.42 ± 2.18
	Phenylpyruvic acid	NADPH	3.70 ± 0.75	2.00 ± 0.59	0.54 ± 0.07
	Hydroxypyruvic acid	NADPH	1.05 ± 0.39	55.59 ± 4.73	57.00 ± 17.74
HPPR3	pHPP	NADPH	0.37 ± 0.06	17.10 ± 0.95	47.07 ± 5.24
	Phenylpyruvic acid	NADPH	1.52 ± 0.05	36.67 ± 4.15	24.09 ± 2.95
	Hydroxypyruvic acid	NADPH	3.57 ± 0.48	3.11 ± 0.21	0.89 ± 0.17
HPPR4	pHPP	Activity was not detectable			
	Phenylpyruvic acid	Activity was not detectable			
	Hydroxypyruvic acid	Activity is minimal (<0.001 μmol min^−1^ mg^−1^)			

*The kinetic parameters of HPR1 were determined with its preferred cofactor, NADH (Timm et al., 2008). For HPPR2 and HPPR3, the kinetic parameters were determined with NADPH (Timm et al., 2008, 2011). Data show means ± SE of three independent experiments.*

### The *HPPR* Genes Encode Cytosolic Proteins and Are Differentially Expressed

**FIGURE 4:**
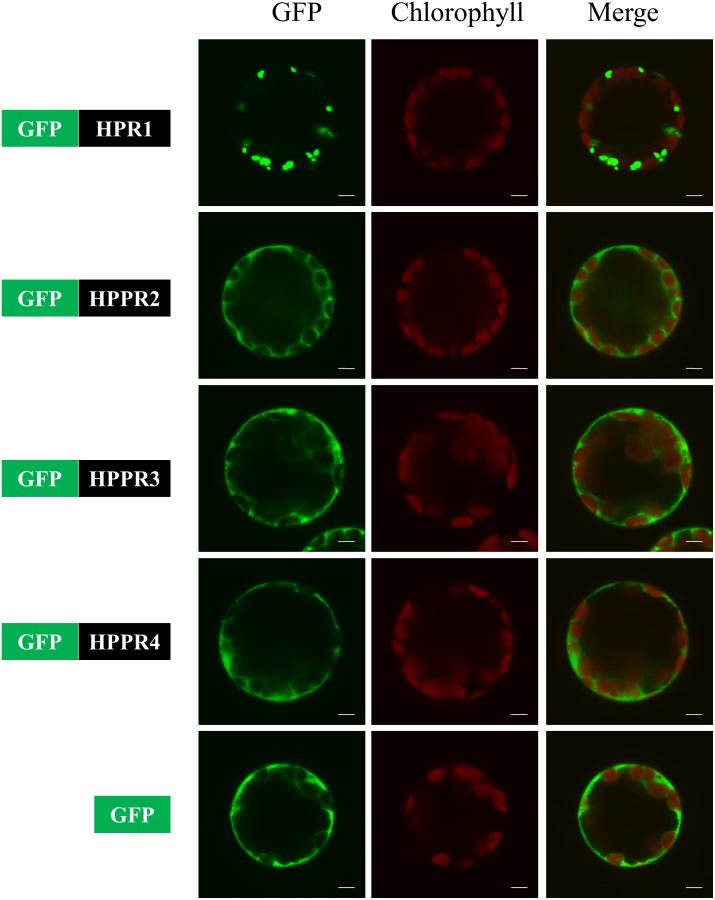
HPPR2, HPPR3, and HPPR4 are localized in the cytosol. The GFP was fused with HPR1, HPPR2, HPPR3, or HPPR4 and transiently expressed in *Arabidopsis* protoplasts. Free GFP was used as a control. GFP fluorescence, chloroplast autofluorescence, and the merged images are shown. Bars = 5 μm.

HPPR2 and HPPR3 were predicted previously to localize in the cytosol and chloroplast, respectively, based on protein sequence analysis (Timm et al., 2008, 2011). To determine their subcellular localizations experimentally, we fused a green fluorescent protein (GFP) to the N-termini of HPR1, HPPR2, HPPR3, and HPPR4. HPR1 has a C-terminal peroxisomal targeting signal-SKL and has already been shown to be a peroxisomal protein (Mano et al., 1997). The chimerical genes were individually expressed in *Arabidopsis* protoplasts from mesophyll cells and fluorescence signals were observed by confocal microscopy. The signals of GFP fused with HPPR2, HPPR3, and HPPR4 were similar to the free GFP signal but distinct from GFP-HPR1, and there was no overlap with chlorophyll florescence for any of the constructs (**Figure 4**). These data suggest that HPPR2, HPPR3, and HPPR4 are cytosolic proteins.

**FIGURE 5:**
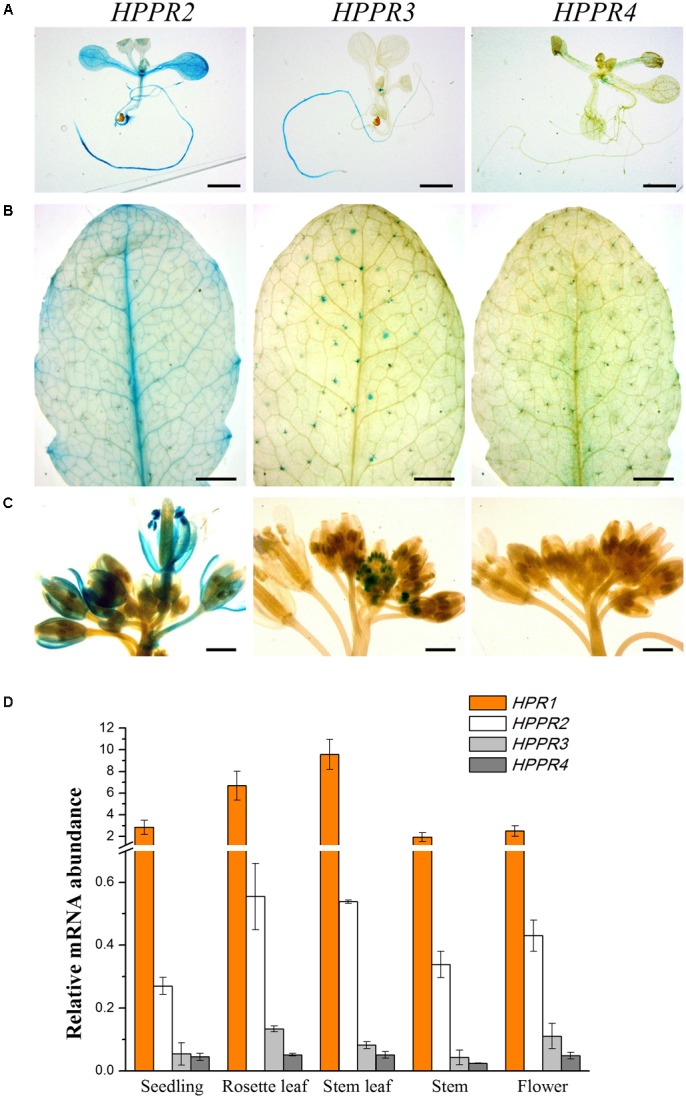
Expression patterns of the *HPPR* genes in plants. **(A–C)**, staining assay of the GUS activities driven by the different *HPPR* gene promoters. **(A)** 9-day-old seedling. **(B)** 4-week-old rosette. **(C)** Inflorescence from a 5-week-old plant. Bars in **(A)** and **(B)** = 2 mm; bars in **(C)** = 1 mm. **(D)** The relative expression levels of *HPR1*, *HPPR2*, *HPPR3*, and *HPPR4* in different organs were analyzed by quantitative reverse transcription PCR (qRT-PCR), with *PP2AA3* (At1g13320) as the internal reference. Data are means of three biological replicates ± SE.

To examine the spatiotemporal expression patterns of the *HPPR* genes, we generated transgenic *Arabidopsis* plants carrying the respective promoter-GUS constructs. In nine-day-old seedlings, *HPPR2* showed a high overall expression, and *HPPR3* was expressed mainly in the root (**Figure 5A**). In rosettes of the four-week-old plants, strong *HPPR2* expression was observed in leaf veins, whereas *HPPR3* expression was restricted to trichomes (**Figure 5B**). In the inflorescence, *HPPR2* was again widely expressed, and *HPPR3* expression was observed in the stamen of young flowers (**Figure 5C**). *HPPR4* expression was weak or below detectable levels (**Figures 5A–C**). Therefore, results from promoter activity suggested that *HPPR2* is broadly expressed throughout the plant whereas *HPPR3* has a more restricted expression pattern.

To determine further the relative expression level, quantitative reverse transcription PCR (qRT-PCR) was performed to analyze the *HPPR* transcripts in seedlings, rosettes, stems, cauline leaves, and flowers (**Figure 5D**). We found that *HPR1* was expressed at considerably higher levels than the *HPPR* genes. Among the three *HPPR*s, *HPPR2* had clearly higher levels of transcripts than *HPPR3* or *HPPR4*.

### HPPR and TAT Defective Mutants Contained Less pHPL

**FIGURE 6:**
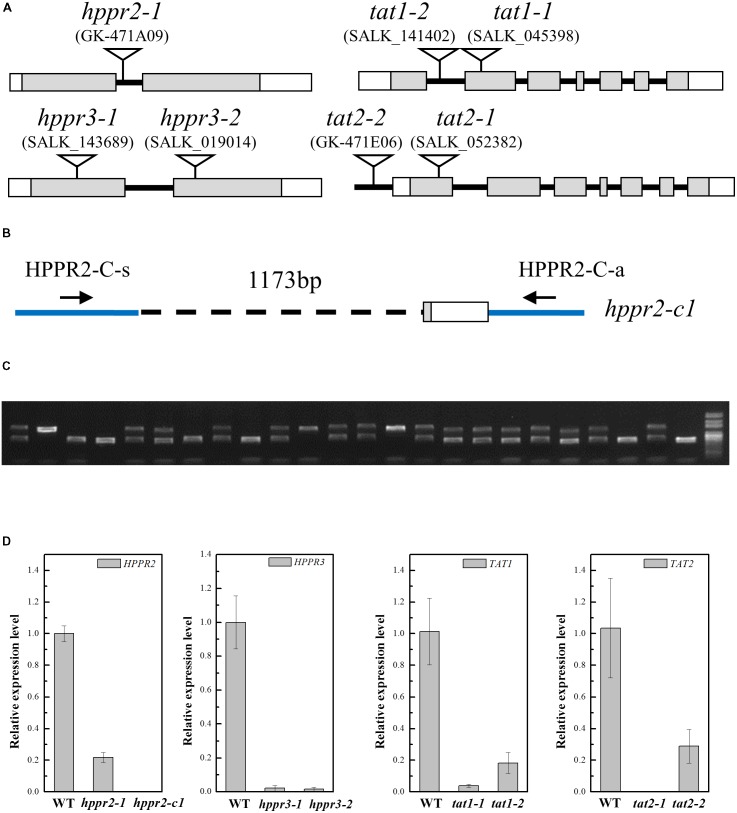
Characterization of the *hppr2*, *hppr3*, *tat1*, and *tat2* mutants. **(A)** Schematic diagram of the T-DNA insertion in *hppr2-1*, *hppr3-1*, *hppr3-2*, *tat1-1*, *tat1-2*, *tat2-1*, and *tat2-2* mutants. Exons and untranslated regions are indicated by gray and white boxes, respectively. **(B)** Schematic diagram showing the *hppr2-c1* mutation, which was generated by CRISPR/Cas9. A 1173-bp fragment was detected in genomic DNA. HPPR2-C-s and HPPR-C-a are the primer pair used to confirm the deletion. **(C)** PCR analysis of DNA from T2 plants generated from a single T1 plant contained the *hppr2-c1* mutation. **(D)** Quantitative reverse transcription PCR (qRT-PCR) analysis of *HPPR2*, *HPPR3*, *TAT1*, and *TAT2* transcript abundance in the wild type (WT) and the T-DNA mutants. Data are means of three biological replicates ± SE.

To provide genetic evidence for the function of *HPPR2* and *HPPR3*, we used three transfer DNA (T-DNA) insertion lines of *A. thaliana*: *hppr2-1*, *hppr3-1*, and *hppr3-2* (**Figure 6A**). In *hppr2-1*, the T-DNA is inserted in the first intron of *HPPR2*, but the two lines of *HPPR3* are knockout mutants (Timm et al., 2011). Homozygous lines were isolated following PCR-based genotyping. In addition, a second allele affecting the *HPPR2* gene, *hppr2-c1*, was generated by CRISPR/Cas9 genome editing using sgRNAs that targeted two separate sites, which deleted a 1173-bp fragment of the *HPPR2* gene (**Figures 6B,C**). In each line, the expression of the target gene was greatly reduced (**Figure 6D**).

TAT catalyzes the first step converting tyrosine to pHPL. We studied T-DNA insertion alleles of *TAT1* and *TAT2*: *tat1-1*, a knockout mutant (Riewe et al., 2012), *tat1-2*, a knockdown mutant with an insertion in the first intron, *tat2-1*, a knockout mutant with an insertion in the first exon, and *tat2-2*, a knockdown mutant with an insertion in the promoter (**Figures 6A,D**).

**FIGURE 7:**
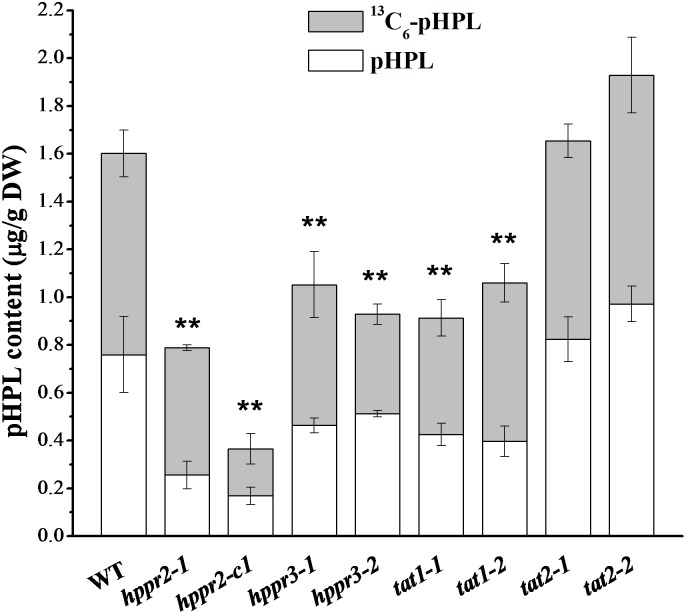
HPPR and TAT are involved in pHPL biosynthesis from tyrosine. Quantification of ^13^C_6_-pHPL and unlabeled pHPL in extracts of 14-day-old seedlings of WT, *hppr2-1*, *hppr2-c1*, *hppr3-1*, *hppr3-2*, *tat1-1*, *tat1-2*, *tat2-1*, and *tat2-2* fed with 250 μM ^13^C_6_-tyrosine for 24 h. Total pHPL contents were analyzed by unpaired two-tailed *t* test compared to WT: ^∗∗^*P* < 0.01. Data are means of three biological replicates ± SE. DW, dry weight.

To determine whether HPPR and TAT together catalyze the formation of pHPL from tyrosine *in planta*, we fed ^13^C_6_-tyrosine to 2-week-old seedlings of wide type and homozygous mutants and analyzed the labeled and unlabeled pHPL by LC-MS/MS (**Figure 7**). We found that pHPL was labeled efficiently with ^13^C atoms in wild-type plants. The *hppr2-1* and *hppr2-c1* mutants displayed substantial decreases (50 to 80%) in labeled and unlabeled pHPL levels compared to wild-type plants, whereas in *hppr3-1* and *hppr3-2* mutants, the pHPL levels were reduced by 30 to 40%. As shown in **Table 1**, HPPR2 is a promiscuous enzyme that has both HPR and HPPR activities, thus as HPPR its catalytic efficiency is relatively low. By contrast, HPPR3 has a strong preference for pHPP and a high HPPR activity. However, *HPPR2* is highly and broadly expressed throughout the plant, while *HPPR3* expression is restricted to specific tissues (**Figure 5**). The difference in their expressions provides an explanation to the observation that pHPL levels were reduced more in *hppr2-1* and *hppr2-c1* mutants than in *hppr3-1* and *hppr3-2* mutants (**Figure 7**).

In *tat1-1* and *tat1-2* mutants, the labeled and unlabeled pHPL levels were ∼60% of that in wild-type plants, whereas no difference was observed between *tat2* mutants and wild type, consistent with the observation that TAT2 has much lower activity toward tyrosine compared to TAT1 (Wang et al., 2016). Together, these results showed that HPPR2, HPPR3, and TAT1 are involved in the biosynthesis of pHPL from tyrosine.

## Discussion

Numerous specialized metabolites are synthesized from tyrosine in plants, yet the enzymes involved in the tyrosine conversion pathways have not been fully identified. In this study, two genes of *Arabidopsis*, *HPPR2* and *HPPR3*, were found to encode proteins that have the apparent HPPR activity: catalyzing the reduction of pHPP to pHPL. The *HPPR2* and *HPPR3* mutants contain less pHPL and are impaired in pHPL biosynthesis. In addition, the knockout mutant of *TAT1* is also compromised in pHPL accumulation. These findings present strong evidence that TAT1, HPPR2, and HPPR3 contribute to pHPL production in *Arabidopsis*.

Interestingly, HPPR is not unique to RA-accumulating plants. Our data clearly indicate that HPPRs are present in the model plant *Arabidopsis*, although RA has not been detected in this species (Petersen et al., 2009). Genes encoding proteins structurally homologous to *HPPR* are found in mosses, ferns, gymnosperms, and angiosperms. This suggests that HPPRs are likely widely distributed in land plants and may represent a general biosynthetic pathway for generating tyrosine-derived metabolites. It is possible that during the evolution of RA biosynthesis, substrates, and enzymes from two conserved metabolic pathways, the tyrosine pathway illustrated here and the phenylpropanoid pathway, were recruited for the formation of a group of specialized phenolics, such as RA in plants of Lamiaceae and Boraginaceae, in which an active RAS is present. In other plants, pHPL may serve as a precursor to different types of natural products besides RA. Phenyllactic acid as well as pHPL have been identified as antifungal metabolites in some strains of lactic acid bacteria (Lavermicocca et al., 2000; Valerio et al., 2004; Mu et al., 2010). Studies in animal systems revealed that pHPL decreases reactive oxygen species (ROS) production in both mitochondria and neutrophils (Beloborodova et al., 2012), thus pHPL itself may function as an antioxidant in plants.

HPPR2 and HPPR3 are phylogenetically distinct from HPR1 (**Figure 2**). HPR1 shows no activity toward pHPP and has strong activity in reducing hydroxypyruvic acid (**Table 1**), which may be excluded from functioning in the tyrosine-related pathways. Another difference between HPR1 and HPPRs involves their subcellular localizations. While HPR1 is a peroxisomal enzyme participating in the photorespiratory core cycle that is essential to organisms with oxygenic photosynthesis, HPPR2 and HPPR3 are responsible for converting tyrosine to phenolic compounds as well as provide a bypass to the photorespiratory cycle (Timm et al., 2008, 2011) in the cytosol (**Figure 4**).

Interestingly, HPPR2 and HPPR3 belong to two phylogenetically distinct groups within the HPPR clade of proteins (**Figure 2**). HPPR2 belongs to group I, which includes proteins commonly found in land plants ranging from *Physcomitrella paten*s and *Selaginella moellendorffii* to seed plants, and HPPR3 is a member of group II which is specific to angiosperms, thus HPPR2 likely evolved earlier. Biochemical characterization of recombinant enzymes shows that, while HPR1 is a specific hydroxypyruvate reductase, both HPPR2 and HPPR3 are promiscuous and HPPR3 is more specialized in reducing pHPP to pHPL. Probably owing to their different expressions patterns, the two enzymes act non-redundantly in the biosynthesis of pHPL (**Figure 7**).

TAT1 and TAT2 are the two well-characterized TAT isoforms of *Arabidopsis* (Prabhu and Hudson, 2010; Riewe et al., 2012; Wang et al., 2016). Kinetic analysis indicated that TAT1 promotes the direction of tyrosine deamination to pHPP, whereas TAT2 promotes transamination of pHPP to tyrosine (Wang et al., 2016). In this investigation, we found that the *tat1* mutants showed a ∼40% decrease in the pHPL levels compared to wild-type plants, whereas the change in the *tat2* mutants was negligible (**Figure 7**). Moreover, TAT1 is localized in the cytosol (Wang et al., 2016), as for HPPR2 and HPPR3 reported herein (**Figure 4**). These findings suggest that TAT1 and the two HPPRs act sequentially in the cytosol to transform tyrosine to pHPL in *Arabidopsis*. In the phenyllactic acid-producing fungal strains, phenylalanine aminotransferase and phenylpyruvate reductase act sequentially in the transformation of phenylalanine to phenyllactic acid (Fujii et al., 2011). Phenylalanine aminotransferase was also identified from rose (Hirata et al., 2012, 2016) and *Cucumis melo* (Gonda et al., 2010). Although phenyllactic acid was not detected from *Arabidopsis*, both HPPR2 and HPPR3 can use phenylpyruvic acid as substrate (**Table 1**). These findings call for further investigations of the distributions of phenylalanine aminotransferase and phenylpyruvate reductase in plants and the pathways in which they are involved.

Taken together, our results show that *Arabidopsis* has two functional HPPRs, HPPR2, and HPPR3, which vary in expression patterns and enzymatic properties, consistent with their phylogenetic divergence. Our findings provide valuable clues for further investigation of metabolic pathways leading to the great diversity of plant phenolics, especially those derived from tyrosine.

## Author Contributions

X-YC, J-JX, LY, CM, and XF wrote the manuscript. X-YC, LY, and J-JX designed the experiments. J-JX performed the experiments. CM helped design some of the experiments. J-JX, XF, X-YC, LY, QZ, and C-YL contributed to the data analysis. All authors discussed the results.

## Conflict of Interest Statement

The authors declare that the research was conducted in the absence of any commercial or financial relationships that could be construed as a potential conflict of interest.
