# Supplementary material for: Characterization of Arabidopsis thaliana Hydroxyphenylpyruvate Reductases in the Tyrosine Conversion Pathway
Source: Front Plant Sci. 2018 Sep 5;9:1305. doi: 10.3389/fpls.2018.01305 (PMC6133988; doi:10.3389/fpls.2018.01305)
Supplement: Supplementary file 1 [file Table_1.DOCX]

**Supplemental Table 1** Primers used in this study

| **Primer Name** | **Sequence (5’ > 3’)** |
| --- | --- |
| **T-DNA mutant genotyping** | |
| GK-471A09-LP | CTTCTTCGCTTCTGGACT |
| GK-471A09-RP | GTGAGGGAGGAGAACTACA |
| pAC161-8474 | ATAATAACGCTGCGGACATCTACATTTT |
| SALK_143689-LP | TCAAAGAGCAGCAGAGGA |
| SALK_143689-RP | TAACTGTGAAAGAGCGAAGA |
| LBb1.3 | ATTTTGCCGATTTCGGAAC |
| SALK_019014C-LP | TAACTGTGAAAGAGCGAAGA |
| SALK_019014C-RP | TCAAAGAGCAGCAGAGGA |
| SALK_045398-LP | CTACCAAAAGCAAGATGACCG |
| SALK_045398-RP | CTTCTTCTTCTTGCGAGTTGC |
| SALK_141402-LP | CTACCAAAAGCAAGATGACCG |
| SALK_141402-RP | CTTCTTCTTCTTGCGAGTTGC |
| SALK_052382-LP | TCAAAACCATCGAGTCCGTAC |
| SALK_052382-LP | CTCTGCAACAGCCCTATTGAG |
| GK-471E06-LP | TCAAAACCATCGAGTCCGTAC |
| GK-471E06-LP | CTCTGCAACAGCCCTATTGAG |
| **qRT-PCR analysis** | |
| HPR1-RT-s | GAACCCGATGTTCCGAGTTG |
| HPR1-RT-a | GATGCTTGGACTGGCATTGG |
| HPPR2-RT-s | AGTGCGATCGCTATGTAAG |
| HPPR2-RT-a | GACATCAGGCTTAATGGTTC |
| HPPR3-RT-s | ACGTCCGGTCTGGTAATTGG |
| HPPR3-RT-a | TCGTCTGTCAAAGAGCAGCA |
| HPPR4-RT-s | CGGTTTTGGCCACTCAAAGG |
| HPPR4-RT-a | ATTTGGCAACCAAAGGCGTC |
| TAT1-RT-s | TGTTTATCACATCGGGTTGC |
| TAT1-RT-a | CCGCAATCTTCATCAAATGCT |
| PP2A-RT-s | ACTCCTCTGGCTAAGCGACT |
| PP2A-RT-a | CGCACCATTGGCATGTCATC |
| TAT2-RT-s | CTGCAACGTTTATTCAGGGA |
| TAT2-RT-a | GCTTGACCTGGTAGGATGAT |
| **CDS gateway cloning** | |
| HPR1-pDonr207-s | GGGGACAAGTTTGTACAAAAAAGCAGGCTTC ATGGCGAAACCGGTGTCC |
| HPR1-pDonr207-a | GGGGACCACTTTGTACAAGAAAGCTGGGTT TCATAGCTTCGAAACAGG |
| HPPR2-pDonr207-s | GGGGACAAGTTTGTACAAAAAAGCAGGCTTC ATGGAATCAATCGGAGTC |
| HPPR2-pDonr207-a | GGGGACCACTTTGTACAAGAAAGCTGGGTT  TCAGACGACCGGAGTCAG |
| HPPR3-pDonr207-s | GGGGACAAGTTTGTACAAAAAAGCAGGCTTC ATGGCGGAATCTTCAGAG |
| HPPR3-pDonr207-a | GGGGACCACTTTGTACAAGAAAGCTGGGTT TCAATCCAATTGAACCGG |
| HPPR4-pDonr207-s | GGGGACAAGTTTGTACAAAAAAGCAGGCTTC ATGCAAAACAGATCAATC |
| HPPR4-pDonr207-a | GGGGACCACTTTGTACAAGAAAGCTGGGTT TCAAAGAACAGGAGTTAA |
| **Promoter gateway cloning** | |
| HPPR2-pro-s | GGGGACAAGTTTGTACAAAAAAGCAGGCTTC GGAGAAGCGCTCTTTCAT |
| HPPR2-pro-a | GGGGACCACTTTGTACAAGAAAGCTGGGTT ATCTCCTTTTCTCTGTTTTTT |
| HPPR3-pro-s | GGGGACAAGTTTGTACAAAAAAGCAGGCTTC GGTAAACGTTCCTCGGCA |
| HPPR3-pro-a | GGGGACCACTTTGTACAAGAAAGCTGGGTT TGTTTTTTTTGTGTGACTCTTC |
| HPPR4-pro-s | GGGGACAAGTTTGTACAAAAAAGCAGGCTTC GCAGGTACGTTCTGATGT |
| HPPR4-pro-a | GGGGACCACTTTGTACAAGAAAGCTGGGTT TATTATTAAAAAATTGTTTTCAAA |
| **Primers used for generating sgRNA plasmids** | |
| HPPR2-sgRNA1-F | TGTGGTCTCAATTGCTTCAACCTTCTTCGCTTCGTTTTAGAGCTAGAAATAGCAAG |
| HPPR2-sgRNA2-F | TGTGGTCTCAATTGTGGGAAATCACTTCTGACTCGTTTTAGAGCTAGAAATAGCAAG |
| sgRNA-R | TGTGGTCTCAAGCGTAATGCCAACTTTGTAC |
| **Primers used to screen mutations at the HPPR2 target** | |
| HPPR2-C-s | CACTGGATTCCCTAAACATGC |
| HPPR2-C-a | CGTGCAACCTACCAAGAAG |
